# Effects of a nurse-led structured home visiting program on quality of life and adherence to treatment in hemodialysis patients

**DOI:** 10.3389/fpubh.2023.1013019

**Published:** 2023-03-09

**Authors:** Mina Pooresmaeil, Sohrab Iranpour, Masoumeh Aghamohammadi

**Affiliations:** ^1^Department of Intensive Care Nursing, School of Nursing and Midwifery, Ardabil University of Medical Sciences, Ardabil, Iran; ^2^Department of Community Medicine, School of Medicine, Ardabil University of Medical Sciences, Ardabil, Iran; ^3^Department of Emergency Nursing, School of Nursing and Midwifery, Ardabil University of Medical Sciences, Ardabil, Iran

**Keywords:** home visit, quality of life, adherence to treatment, hemodialysis, nurse

## Abstract

**Purpose:**

This study aimed to determine the effects of a nurse-led structured home visit program on quality of life and adherence to treatment in patients undergoing hemodialysis.

**Methods:**

The study was quasi-experimental research in which 62 hemodialysis patients referred to Bu Ali hospital in Ardabil participated in two groups: Intervention (*n* = 31) and control (*n* = 31). The intervention included a structured and planned home visit program that was performed in five stages over 3 months. Data collection tools were a demographic information form, Kidney Disease Quality of Life Short Form (KDQOL–SF™) and End Stage Renal Disease Adherence Questionnaire (ESRD_AQ) which were completed by patients before, at the end of the first, second, and third month of intervention. SPSS v20 software and descriptive and analytical tests (Chi-square, *t*-test, ANOVA and repeated measure) were used for data analysis.

**Findings:**

Examining demographic characteristics showed that there is a negative and significant relationship between age and quality of life scores (*P* = 0.004), that is, with increasing age, the quality of life score decreases, but other demographic characteristics did not have a significant relationship with quality of life scores and adherence to treatment (*P* > 0.05).

Also, the results showed that in the intervention and control groups, during the study, the scores of quality of life and adherence to treatment increased significantly, and this increase was significantly higher in the intervention group than in the control group (*P* < 0.001).

The scores of quality of life and adherence to treatment increased significantly both during the study in each group separately and between groups during the study (*P* < 0.001).

**Conclusions:**

According to the significant improvement in quality of life and adherence to treatment in patients following a home-visiting program during 3 months, these interventions can be utilized to improve quality of life and adherence to treatment of patients undergoing hemodialysis.

**Practice implications:**

Home visiting programs significantly improve the level of knowledge of patients undergoing hemodialysis and their family members, through their involvement in the care process. Having said that, it seems plausible to implement home visits in the standard care plans of hemodialysis patients.

## Introduction

End-Stage Renal Disease (ESRD) is characterized by a glomerular filtration rate of < 15 ml/min. At this stage, various clinical manifestations such as hypertension, anemia, edema, metabolic disorders, and endocrine disorders may occur that require renal replacement therapy such as hemodialysis (HD) (1).

The life of these patients changes due to changes in diet, frequent use of nutritional supplements, fluid restriction and multiple dialysis sessions. Due to the lifestyle changes and treatment, these patients often experience Physical and mental problems (2), all of which can lead to a lower quality of life (QOL) (3). Quality of life (QOL) is considered an important issue in evaluating the outcomes of patients receiving health care. Although there is no consensus on the definition of quality of life, it has been found that in patients with kidney failure, especially in patients undergoing dialysis, QOL affects more physical aspects and less mental functioning (4). It is important to pay attention to the quality of life of these patients because, according to some evidence, it is related to medical outcomes, including the reduction of hospitalization and mortality due to hospitalization (5, 6).

Non-adherence to treatment is also one of the main clinical concerns in patients undergoing hemodialysis (7). Adherence to treatment which is defined as the degree to which individuals' behavior conforms to health or treatment recommendations, is a complex behavioral process and is influenced by several factors, such as patients' personal characteristics, physician-patient interactions, and the quality of the health care system (8).

Poor adherence or non-adherence of patients to treatment is one of the main reasons for failure in a treatment plan, increased complications, prolongation of treatment, and increased healthcare costs (9). According to reports, ~25–86% of hemodialysis patients do not follow their treatment regimen (10, 11). The study conducted by Gerogianni et al. showed that rejection of treatment and treatment limitations were among the most important problems of hemodialysis patients (12). Furthermore, polypharmacy and the inability to purchase all the required drugs are among the notable problems (13).

Moreover, many patients report feelings of anger, guilt, and fear about their illness, and most of them have no motivation to take care of themselves and adhere strictly to treatment (14). Therefore, due to the rising trend of chronic renal failure and the prevalence of physical and mental problems in hemodialysis patients and the resulting complications and consequences, the existence of effective interventions as a crucial element in the treatment of these patients is essential (15).

One way to provide care is home visits. During home visit, the patients and their family members are educated on the healthcare needs of their patient in the home environment in order to allow them to meet these needs independently. Home is an intimate environment for the patient and their family members to interact with the nurse, and in some cases a home visit is the only way to access information or to educate, reduce health risks, promote health, and provide services to families (16). Home visits allow the health professionals to identify the health problems of the patients, and when necessary, set treatment plans in order to improve their quality of life (17). In addition, the home environment allows for a more realistic assessment, an efficient identification of the risk factors and problems, and the initiation of the interventions in the early stages (18).

There only a small number of studies that have investigated the effect of home visiting programs and their effect on quality of life in particular including the study of Liimatta et al. titled “The effect of home visit on the quality of life of the elderly” (19). Ahangarzadeh Rezaei et al. (20) also investigated the effect of home visiting programs on improving the physical condition of hemodialysis patients and considered it as a basic yet important method in the healthcare (20).

In addition, home visits may provide unique opportunities to identify and address issues that may exacerbate the illness. In the home visiting program, a caregiver may collect vital information about following up on patients' medical visits and how to take medication. Educating patients and their families about medical treatment events, managing acute or chronic conditions, and detecting warning signs of illness are among the other advantages of a home visit program (21).

Despite the rising number of patients requiring hemodialysis and the importance of their education by nurse practitioners, the effect of home visiting programs on quality of life and treatment adherence has not been widely studies in these patients. To that end, the present study aims to determine the effect of a nurse-led structured home visiting program on quality of life and treatment adherence in patients undergoing hemodialysis in Ardabil, Iran.

## Methods

### Study design

The present study was a quasi-experimental research with a control group.

### Participants

The study population was patients undergoing hemodialysis referred to Bu Ali Hospital and the Red Crescent Center of Ardabil in 2021. Inclusion criteria included patients aged 18–65 years undergoing hemodialysis, with a history of dialysis for more than 6 months and at least twice a week, no history of known mental disorders, no hearing problems, no history of formal education in the last year, and willingness to participate in the study. Change of residence during the study, having a kidney transplant surgery, and cessation of hemodialysis were regarded as exclusion criteria. Sample size was calculated based on the statistical formula

$$\begin{array}{l} n\;=\;\frac{{\left(z_{1-\alpha }+z_{1-\beta }\right)}^{2*}\left({s_{1}}^{2}+{s_{2}}^{2}\right)}{{\left({\bar{X}}_{1}-{\bar{X}}_{2}\right)}^{2}} \end{array}$$

**Figure 1:**
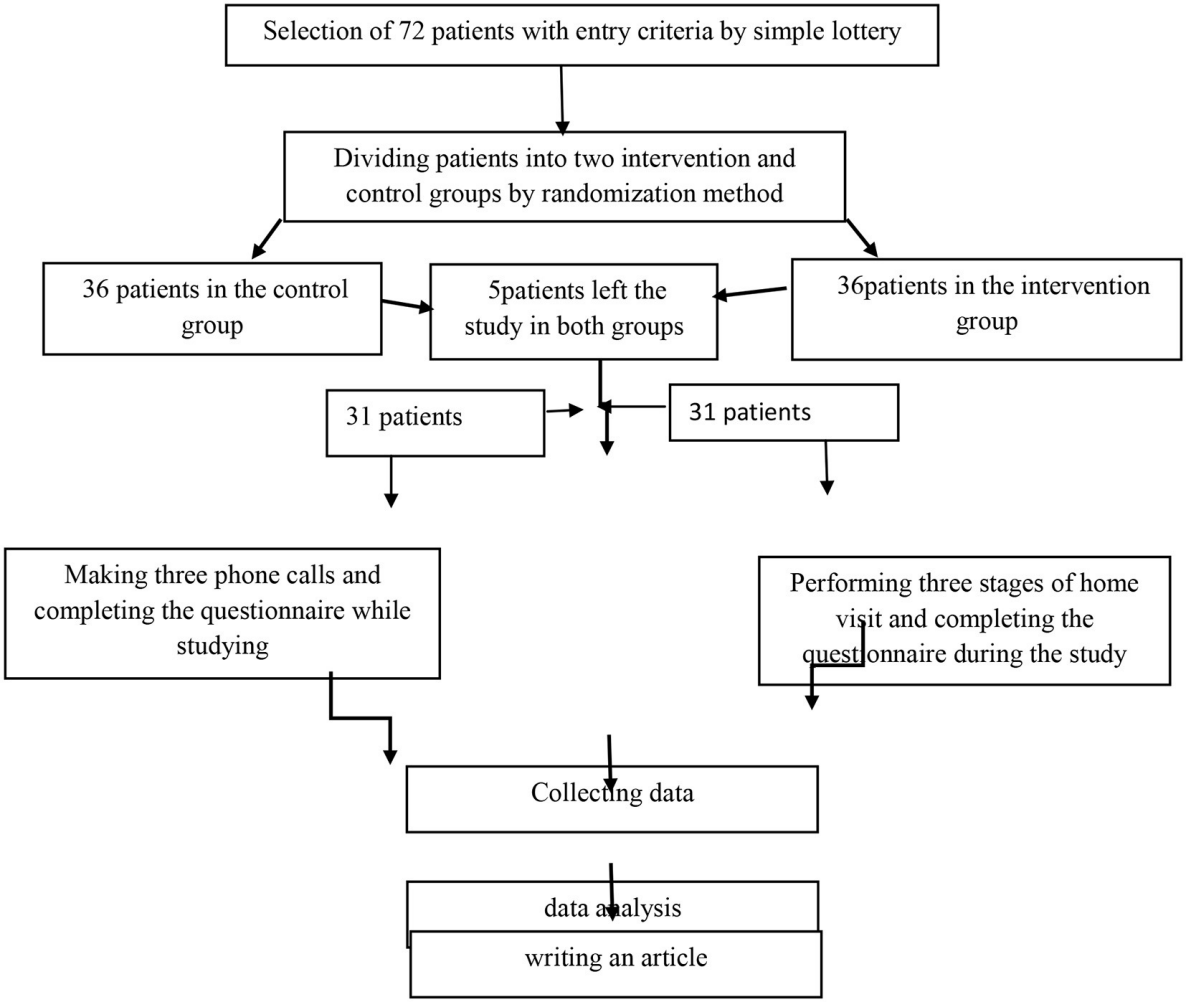
Study flowchart.

- α: Probability of first type error; If α = 0.05, Z1-α/2 is equal to 1.96.
- β: Probability of second type error; If β = 0.02, Z1-β is equal to 1.96.
- σ_1_: The standard deviation of the trait in the first community
- σ_2_: Standard deviation of the attribute in the second society
- X_1_: Average trait in the first community

and the results of (22) and considering α = 0.05 and β = 0.02, the test power of 80, and the possible fall of 72 patients (36 people in the intervention group and 36 people in the control group). The patients were initially sampled randomly (through a lottery system) and were assigned to the intervention and control groups using the permuted block randomization method (Figure 1).

In this study, 6 blocks of 4 were used to randomly assign patients to two intervention and control groups. The intervention group was named A and the control group was named B, and the following 4 conditions were created in each block:

Next, having six hypothetical blocks, six numbers (1 to 6) were used for random selection. Seventy two patients were coded after 18 random selections of blocks of 4. Based on the initial estimate of the required number of samples (*n* = 72) and the two required groups, 72 codes were prepared, 36 codes of the control group and 36 codes of the intervention group were written.

After receiving the code of ethics from the Ethics Committee of Ardabil University of Medical Sciences, obtaining written consent from the patients, and assuring them that their information was not disclosed, the intervention began. The intervention was the home visit of hemodialysis patients based on home visit model (23). This intervention consisted of several steps as follows:

Initial stage: In this stage, after selecting the samples based on the entry criteria and randomization, informed consent was obtained from the samples and then the objectives of the research were explained.

Pre-visit stage: In this stage, the duration of hemodialysis, the time of hemodialysis during the day, the drugs received by the patients during dialysis and at home, the amount of ultrafiltration that is reduced on average from the patient during dialysis, the weight of the patients, the settings that are given to the dialysis machine such as sodium, temperature, etc…, the type of vascular access of the patient and how it works, the medical orders in the file, the history of the patient's previous hospitalizations in other medical centers, the problems that arise for the patient during dialysis under the dialysis machine, and finally, the patient's intolerance or non-cooperation during dialysis It was obtained from the clinical records of patients in hemodialysis centers. At this stage, an appointment was also made with the opinion of the clients.

At-home stage: First, the researcher introduced himself to the patient and family members. Again, about the home visit, the objectives of the study were discussed with the patients and their families. Then, in the first visit, all the questionnaires were filled before the start of the intervention. After filling the questionnaires, the training of the patients started. Our training included all aspects of the quality of life and adherence to the treatment Also, about the physiology of the disease, the process of hemodialysis, access to dialysis and related care, education about diet and fluid intake, important points about drugs, activity level, problems of hemodialysis patients such as itching, muscle cramps, depression, disorders Sleep and etc. The educational needs of the patients were discussed for 45–60 min and the questions of the patient and the family were answered.

Quality of life and Adherence to treatment questionnaires are always filled before communicating with patients at this stage.

Final stage: At this stage, the educational materials were summarized and the evaluation of the learned items was summarized. Also, an educational booklet was given to the patients. The samples were followed up by phone for 1 month.

Post-visit stage: A report of the activities performed during the visit was made and a planning was made for the next visit. Finally, the questionnaires were scored and entered into the software SPSS.

A monthly home visit was conducted for three consecutive months for the intervention group, and the necessary explanations were provided based on the educational needs of each patient. The patients could also call the researchers with their inquiries before the time of the visit.

In the control group, after obtaining written and informed consent, the purpose of the study was shared with the patient and family members. Afterward, the patients' medical records were studied, and the patient or their main caregiver was contacted every month. Additionally, based on the patients' educational needs, the required explanations were provided. In case of any inquiries, the patients could contact the researchers.

### Data collection

Evaluations were conducted for each patient in the 4 stages including before the intervention, at the end of first, second and third month based on demographic information form (age, sex, marital status, occupation, level of education, medical history, duration of dialysis, and occupational status), Kidney Disease Quality of Life- Short Form (KDQOL–SF™ 1.3), and End Stage Renal Disease- Adherence to Treatment Questionnaire (ESRD_AQ). The demographic information questionnaire was completed only once in the first session, and the next two questionnaires were completed in each of the four sessions. The KDQOL-SF instrument is a standardized self-report instrument that includes 8 health-related quality of life subscales and 11 kidney disease-specific quality of life subscales. The tool of health-related quality of life, which is the general core of KDQOL-SF, is the same 36-question questionnaire (SF-36). This tool has 8 dimensions of physical performance (10 questions), role limitation caused by physical problems (4 questions), role limitation caused by emotional problems (3 questions), social function (2 questions), emotional well-being (5 questions), examines pain (2 questions), fatigue and energy (4 questions), understanding of general health (5 questions) and a general question about personal health. The second part of the KDQOL-SF instrument focuses on health-related items of people with kidney disease and undergoing hemodialysis, which is divided under the title of Kidney Disease Component Summary (KDCS) and includes subscales: symptoms (signs/problems); 12 questions), the effect of kidney disease on life (8 questions), burden of responsibility for kidney disease (4 questions), job status (2 questions), cognitive function (3 questions), quality of social interaction (3 questions), sexual function (2 question), sleep (4 questions), social support (2 questions), encouragement by dialysis department staff (2 questions) and patient satisfaction (1 question). To answer this questionnaire, multiple-choice Likert is used, which is assigned a score from zero to 100 for each dimension. Higher scores indicate a better quality of life. The results of the study conducted by Yekaninejad et al. (24) indicated high internal consistency on all scales (range of alpha-Cronbach coefficients from 0.73 to 0.93) (24).

A self-report questionnaire of treatment adherence behaviors among patients with end-stage renal disease (ESRD-AQ) was developed by Kim (25). This 46-item questionnaire is designed for patients needing dialysis treatment and has five sections. The first section examines general information about the patients with end-stage renal disease (5 items), and the remaining four sections, namely attendance at sessions (14 items), medication adherence (9 items), fluid restriction (10 items), and diet (eight items), evaluates treatment adherence in hemodialysis patients. The total score of treatment adherence is the sum of the scores of these five sections. The lowest score of the questionnaire is zero, and the highest score is 1,200. Khalili et al. (26) first psychometrically assessed this tool in Iran and the questionnaire was found to be valid. The reliability of the instrument was also confirmed by the Cronbach's alpha coefficient of 0.75 (26).

### Statistical analysis

Data analysis was conducted using SPSS version 25. Descriptive statistics were used to evaluate the samples' demographic characteristics The relationship between demographic characteristics and quality of life scores and adherence to treatment in the intervention and control groups was investigated with linear regression tests, *t*-test and analysis of variance. The Kolmogorov-Smirnov test was performed to evaluate data normality. To achieve the research objectives, descriptive statistics methods (mean, standard deviation, frequency, and percentage), *t*-test, Chi-squared test, and repeated measures analysis of variance (ANOVA) were performed. The significance level was considered <0.05.

## Results

### Participants' demographic characteristics

Owing to the exclusion of 10 participants from the study (7 patients reluctant to continue cooperation, 1 patient due to change of residence, and 2 patients due to death), this study was conducted on 62 patients undergoing hemodialysis (31 patients in the intervention group and 31 patients in the control group). Table 1 presents the demographic information of the studied patients. As the table shows, the patients' mean age and standard deviation in the intervention and control groups were 48.70 ± 13.98 and 54.38 ± 8.57 respectively. The minimum age of the participant was 22 and the maximum was 64. There was no significant difference between intervention and control group regarding the demographic characteristics (*P* > 0.05).

**Table 1:** Demographic characteristics of patients in the intervention and control groups.

**Group**		**Intervention**		**Control**		**Chi-square test results**
**variable**		***N***	**%**	***N***	**%**	
Gender	Male	12	38.7	17	54.8	0.15 = P
	Female	19	61.3	14	45.2	
Marital status	Married	24	77.4	24	77.4	0.29 = P
	Single	6	19.4	1	3.2	
	Widow	1	3.2	5	16.1	
	Divorced	0	0	1	3.2	
Job	Unemployed	10	32.3	7	22.6	0.32 = P
	Worker	0	0	1	3.2	
	Employee	1	3.2	1	3.2	
	Housework	16	51.6	13	41.9	
	Freelance worker	4	12.9	9	29	
Level of Education	High school	17	54.8	24	77.4	0.09 = P
	Diploma	11	35.5	6	19.4	
	Associate Degree	1	3.2	0	0	
	Bachelor's degree and higher	2	6.5	1	3.2	
Disease background	Yes	27	87.1	25	80.6	0.36 = P
	No	4	12.9	6	19.4	
Age (mean ± SD)		48.70 ± 13.98		54.38 ± 8.57		*P =* 0.34^*^

* Independent sample T-test.

### The effect of home visiting program on the subscales and two main dimensions of quality of life

The mean and standard deviation were calculated in all the subscales of the quality of life questionnaire. In most cases, with the progress of the study, a statistically significant difference was observed in the intervention and control groups (*P* < 0.05), except for the subscales Work status (*P* = 0.43), Cognitive function (*P* = 0.70) and Physical functioning (*P* = 0.41) where the relationship between the intervention and control groups was not significant (Table 2).

**Table 2:** Quality of life (subscales) in the intervention and control groups during the intervention.

**Dedicated dimension**		**Before intervention**	**The first month**	**The second month**	**The third month**	***F***	**P-value**
**(SF-36)**		**Mean** ±**SD**	**Mean** ±**SD**	**Mean** ±**SD**	**Mean** ±**SD**		
Symptom/problem list	Intervention group	65.79 ± 5.55	71.23 ± 5.22	76.07 ± 5.15	76.41 ± 5.33	57.25	<0.001
	Control group	58.53 ± 5.44	61.76 ± 5.60	64.58 ± 6.01	65.67 ± 6.45		
Effects of kidney disease	Intervention group	49.97 ± 10.77	56.55 ± 10.02	60.28 ± 9.95	63.20 ± 10.26	17.91	<0.001
	Control group	44.55 ± 10.04	46.67 ± 9.94	48.28 ± 8.41	50.30 ± 9.28		
Burden of kidney disease	Intervention group	39.91 ± 8.64	64.91 ± 11.02	72.58 ± 9.50	77.82 ± 10.56	185.95	<0.001
	Control group	36.29 ± 7.64	39.51 ± 7.28	42.54 ± 7.29	44.75 ± 6.27		
Work status	Intervention group	12.90 ± 28.77	32.25 ± 35.46	32.25 ± 35.46	32.25 ± 35.46	0.623	0.433
	Control group	12.90 ± 25.71	17.74 ± 30.40	24.19 ± 33.84	30.64 ± 35.77		
Cognitive function	Intervention group	32.25 ± 9.28	29.24 ± 9.05	29.24 ± 9.05	29.24 ± 9.05	0.140	0.709
	Control group	27.52 ± 8.90	29.89 ± 10.16	32.68 ± 9.94	33.33 ± 10.32		
Quality of social	Intervention group	49.24 ± 8.37	46.45 ± 7.97	46.45 ± 7.97	46.45 ± 7.97	5.66	0.002
	Control group	42.58 ± 9.98	46.23 ± 10.31	47.95 ± 8.50	47.95 ± 8.50		
Sexual function	Intervention group	60.88 ± 23.21	68.14 ± 20.37	68.54 ± 20.37	72.17 ± 18.17	3.44	0.06
	Control group	54.83 ± 17.28	58.46 ± 15.93	60.88 ± 15.72	62.50 ± 14.43		
Sleep	Intervention group	65.00 ± 6.48	70.56 ± 6.44	75.08 ± 7.65	81.20 ± 7.32	44.97	<0.001
	Control group	59.19 ± 8.59	60.00 ± 9.21	61.45 ± 8.48	62.09 ± 8.44		
Social support	Intervention group	81.71 ± 13.16	89.24 ± 11.82	93.00 ± 9.40	95.69 ± 8.57	8.210	0.006
	Control group	76.34 ± 17.62	79.02 ± 16.65	82.25 ± 17.18	86.01 ± 14.33		
Dialysis staff encouragement	Intervention group	88.30 ± 30.00	91.53 ± 8.15	93.54 ± 7.82	96.77 ± 5.56	7.35	0.009
	Control group	85.48 ± 9.18	87.09 ± 9.40	88.30 ± 8.49	89.91 ± 8.79		
Patient satisfaction	Intervention group	94.62 ± 7.92	95.69 ± 7.41	98.38 ± 5.01	98.92 ± 4.16	8.412	<0.001
	Control group	92.47 ± 8.43	94.08 ± 8.10	95.16 ± 7.69	95.69 ± 4.16		
**General dimension (KDCS)**							
Physical functioning	Intervention group	53.06 ± 10.05	56.81 ± 9.03	59.03 ± 8.60	61.12 ± 7.71	0.672	0.416
	Control group	51.29 ± 7.74	57.34 ± 7.77	59.35 ± 6.79	55.64 ± 11.08		
Role physical	Intervention group	4.83 ± 10.04	28.22 ± 23.04	70.56 ± 23.36	48.38 ± 29.53	8.103	0.006
	Control group	12.90 ± 16.92	12.90 ± 16.92	19.35 ± 21.12	21.77 ± 27.78		
Pain	Intervention group	38.30 ± 12.04	60.48 ± 17.99	70.56 ± 15.99	81.04 ± 12.44	86.53	<0.001
	Control group	30.64 ± 15.75	33.06 ± 14.98	35.48 ± 13.34	37.09 ± 12.70		
General health	Intervention group	31.29 ± 6.32	52.74 ± 8.54	62.25 ± 8.54	70.48 ± 10.25	136.947	<0.001
	Control group	29.51 ± 6.37	34.51 ± 6.99	39.03 ± 7.89	42.90 ± 7.72		
Emotional well-being	Intervention group	50.58 ± 6.97	59.87 ± 6.65	67.22 ± 6.31	74.58 ± 6.24	78.89	<0.001
	Control group	43.09 ± 7.56	46.19 ± 8.38	49.03 ± 10.01	51.74 ± 10.11		
Role emotional	Intervention group	12.90 ± 26.77	20.10v29.00	33.33v28.54	36.55 ± 27.69	56.83	<0.001
	Control group	22.58 ± 21.75	25.80 ± 23.89	29.03 ± 28.20	30.10 ± 27.69		
Social function	Intervention group	38.30 ± 9.64	54.43 ± 14.98	64.51 ± 16.48	69.75 ± 16.38	33.00	<0.001
	Control group	35.08 ± 13.07	38.70 ± 13.44	40.72 ± 14.05	42.33 ± 12.36		
Energy/fatigue	Intervention group	34.83 ± 6.89	45.80 ± 8.27	55.80 ± 7.75	68.06 ± 8.13	30.22	<0.001
	Control group	36.45 ± 9.59	38.87 ± 9.37	42.74 ± 8.44	47.09 ± 9.01		

Quality of life scores were calculated in two main dimensions (general and specific). To analyze the data, ANOVA was used. The studies showed that the scores of the quality of life in the intervention and control groups in both general and specific dimensions increased significantly during the intervention, and this increase was more in the intervention group than in the control group (Table 3).

**Table 3:** Quality of life (Dimensions) and adherence to the treatment in the intervention and control groups during the intervention.

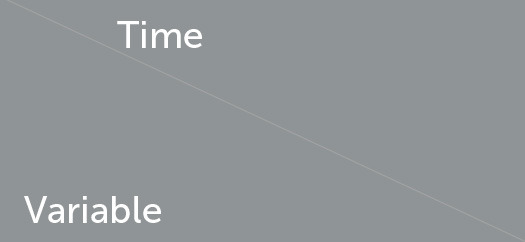		**Before intervention**	**The first month**	**The second month**	**The third month**	F (*P*-value^*^) Intergroup comparison	F (*P*-value^*^) Interactive effect (group and time)
		**Mean** ±**SD**	**Mean** ±**SD**	**Mean** ±**SD**	**Mean** ±**SD**		
Quality of life	Intervention group	35.29 ± 2.18	45.47 ± 2.60	49.43 ± 2.69	52.64 ± 2.77	147.761 (< 0.001)	131.729 (< 0.001)
	Control group	37.83 ± 2.73	37.85 ± 2.39	40.04 ± 2.90	41.08 ± 3.60		
Specific dimension of quality of life	Intervention group	56.65 ± 4.14	63.56 ± 4.18	67.56 ± 4.08	66.00 ± 4.74	94.377 (< 0.001)	26.150 (< 0.001)
	Control group	51.30 ± 2.62	54.00 ± 3.23	56.42 ± 3.51	56.52 ± 4.05		
General dimension of quality of life	Intervention group	36.87 ± 4.37	50.00 ± 4.25	56.92 ± 5.11	63.24 ± 5.51	83.312 (< 0.001)	105.210 (< 0.001)
	Control group	36.34 ± 4.471	4.25 ± 4.17	43.47 ± 5.61	44.41 ± 7.43		
Adherence to treatment	Intervention group	767.74 ± 155.88	900.80 ± 112.45	1,026 ± 104.66	1,076.61 ± 99.14	13.732 (< 0.001)	3.305 (0.02)
	Control group	694.18 ± 118.23	897.58 ± 101.03	975.00 ± 84.90	1,018.54 ± 61.90		

* ANOVA.

### The effect of home visiting program on quality of life

The results of the ANOVA indicated a statistically significant difference between the experimental and control groups in terms of changes in the mean score of quality of life in the previous 4 stages until the end of the third month of the intervention (*P* < 0.05). The mean scores of quality of life in the intervention group in the pre-intervention stage, the end of the first, second, and third month were 35.29 ± 2.18, 45.47 ± 2.60, 49.43 ± 2.69, and 52.64 ± 2.77, respectively; this upward trend was significant (p < 0.05). Also, the change in the mean scores in 4 stages, before the intervention (37.83 ± 2.73), the end of the first (37.85 ± 2.39), the second (40.04 ± 2.90), and the third month (41.08 ± 3.60), was significant in the control group (Table 3).

**Figure 2:**
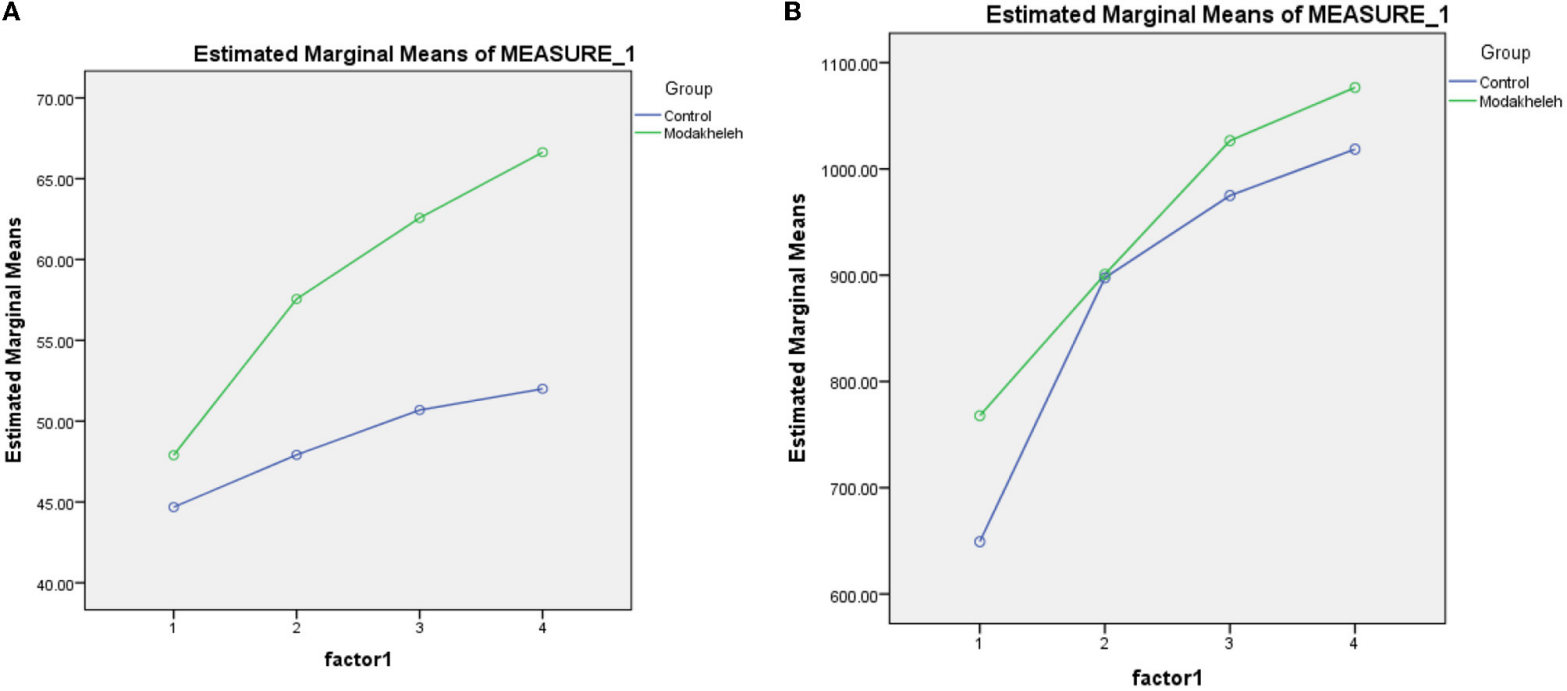
**(A)** Changes in the quality of life during the intervention process in both groups. **(B)** Changes in the adherence to treatment during the intervention process in both groups.

Figure 2A shows the changes in total quality of life scores during the study in both groups.

### The effect of home visiting program on treatment adherence

According to Table 3 and the results of the ANOVA, the average score of adherence to treatment in patients of the intervention group in the time intervals before the intervention (767.74 ± 155.88), in the first month (900.80 ± 112.45) in the end of the second month (1026 ± 104.66) and in the end the third month of the intervention (1,076.61 ± 99.14) had a significant increase (*P* < 0.05), in the control group, the score between before and 3 month after the intervention showed a significant change (*P* < 0.05). These results were the result of ANOVA showing that the upward trend of treatment adherence scores in the intervention group was significant compared to the control group (*p* < 0.05).

Figure 2B shows the changes in total treatment adherence scores during the study in both groups.

Table 3 also shows that the scores of quality of life and adherence to treatment increased significantly both during the study in each group separately and between groups during the study.

Statistical analysis showed that there is a significant relationship between age and quality of life in both the intervention and control groups (*p* < 0.05), In a way that the quality of life decreases with increasing age. There was no significant relationship between other demographic characteristics in the intervention and control groups with quality of life and adherence to treatment (*p* > 0.05).

## Discussion

The findings of this study showed a significant improvement in the quality of life of patients from an unfavorable level in the pre-intervention period to a high level at the end of the third month of the intervention. Therefore, it appears that the use of a well-codified and planned home visiting program can be effective in improving the quality of life of patients undergoing hemodialysis. In this regard, studies conducted on patients with schizophrenia (27), type II diabetes (28–30), burns (31), hypertension (30) and on couples with stress and anxiety (32) reported similar findings. This suggests that home visiting programs can motivate patients to take responsibility for their treatment by actively involving them in the treatment process. Additionally, an effective, one-on-one, and dynamic relationship can be established between the patient and the nurse practitioner which allows for a better understanding of the patients' needs and problems and the nurses' expectations. This improves patient adaptation through the development of self-care and problem-solving skills, thereby playing a crucial role in the individuals' quality of life.

Furthermore, the results of the present study on the effect of home visiting intervention on treatment adherence of hemodialysis patients showed a significant increase in the mean score of treatment adherence of patients in the intervention group at the end of the second and third months of the intervention. This finding is in line with the results of the studies conducted by Lockwood et al. (33) on patients with hospital-acquired discharge pelvic fractures, Comulada et al. (34) on patients with acquired immunodeficiency infection, Justvig et al. (35) on elderly patients with hypertension, and Chow et al. (36) on patients with diabetes (33–37). One of the important factors influencing the treatment adherence of patients with chronic diseases is to raise their level of awareness to increase the acceptance of treatment and its continuation (38). It appears that a home visiting program, such as the one implemented in this study, can successfully improve treatment adherence by raising the level of awareness of the patients. Moreover, considering the relationship between quality of life and treatment adherence, the improved treatment adherence of the patients during the 3 months of home visit intervention can be related to the patients' increased quality of life.

## Limitations

The present study was conducted only on hemodialysis patients in Ardabil, Iran; therefore, its results cannot be generalized to all patients undergoing hemodialysis. Accordingly, it is suggested that the effect of this care model needs to be examined on the abovementioned variables and evaluated with a larger sample size.

Another limitation of our study was the failure to calculate the cost of the intervention and its cost-effectiveness, so it is suggested to calculate the cost of the intervention for future studies.

## Conclusions

The results of this study demonstrate that a nurse-led structured home visiting program significantly improved the quality of life and treatment adherence in hemodialysis patients during the 3 months after the intervention. Although, the cost of the home visit in the intervention group has not been investigated in the study, in several studies conducted on the benefits of home visit plans, the issue of reducing costs has been significantly mentioned (39–41), It is therefore recommended to implement this program in the standard care plan of hemodialysis patients by informing them about their disease and reinforcing self-care according to the facilities and conditions of the home environment.

## Data availability statement

The original contributions presented in the study are included in the article/supplementary material, further inquiries can be directed to the corresponding author.

## Ethics statement

The studies involving human participants were reviewed and approved by the Ethics Committee in Biomedical Research at Ardabil University of Medical Sciences (ARUMS) (ethicIR.ARUMS.REC.1400.065). The patients/participants provided their written informed consent to participate in this study.

## Author contributions

MP and MA designed the study and had a role in preparing the manuscript. MP held home visit sessions and collected the data. MP and SI analyzed the data. All authors approved the final manuscript.
